# Alopecia and Mental Health: Integrating Dermatological and Psychiatric Perspectives in Adolescence

**DOI:** 10.1192/j.eurpsy.2026.11235

**Published:** 2026-07-31

**Authors:** E. López Bardón, M. M. Menéndez Muñoz, C. P. Carbonel Alonso, J. Gómez Ruiz, C. M. Martinez

**Affiliations:** Complejo Asistencial Universitario de León, León, Spain

## Abstract

**Introduction:**

Alopecia in adolescence often has a strong psychological component and may act as both a trigger and a perpetuating factor of psychiatric symptoms. The psychosocial burden of visible hair loss is particularly pronounced during adolescence, when body image, self-esteem, and social integration are highly vulnerable. This case highlights the interaction between dermatological symptoms and psychiatric manifestations in a young woman whose alopecia was closely linked to anxiety and depressive symptomatology

**Objectives:**

To evaluate the clinical course and multidisciplinary management of a patient with alopecia and comorbid anxiety and depressive disorder, emphasizing the importance of an integrative approach

**Methods:**

Case report based on retrospective review of psychiatric and dermatological records, complemented by clinical observation and long-term follow-up

**Results:**

We present the case of a 22-year-old woman, in psychiatric follow up since the age of 15 for reactive anxiety and depressive symptoms secondary to alopecia. Dermatological evaluation revealed no organic causes or significant trichological abnormalities, and multiple treatment attempts failed to improve hair loss. Psychological assessment identified relevant stressors including family conflict, religious pressure, and lack of peer relationships. At age 17, she attempted suicide following family rejection of her partner, in the context of academic underperformance. Notably, partial hair regrowth was observed during periods of family stability and school holidays.

Family history revealed a paternal uncle who died by suicide, several maternal aunts under psychiatric care for depression, and a younger brother presenting alopecic plaques. On examination, the patient showed depressive mood, emotional lability, irritability, apathy, anhedonia, and somatic and cognitive anxiety with frequent rumination on physical appearance. Personality traits included high self-demand and anankastic features. No psychotic symptoms or current suicidal ideation were detected.

Treatment with paroxetine (20 mg/day) and lorazepam (1 mg/12 h, with additional doses as needed) was initiated. After three months, significant improvement in anxiety and depressive symptoms was noted, without new alopecic plaques. At six months, a sustained favorable evolution allowed dermatology to discharge the patient.

**Conclusions:**

Alopecia may function as both a trigger and a perpetuating factor of anxiety and depressive symptoms in adolescence, significantly affecting self-esteem and social Multidisciplinary management is essential in cases where organic, psychological, and family factors converge. Combined psychopharmacological treatment, psychotherapy, and improvement of family dynamics contributed to both psychiatric stabilization and dermatological recovery

**Disclosure of Interest:**

None Declared

